# Understanding Host-Switching by Ecological Fitting

**DOI:** 10.1371/journal.pone.0139225

**Published:** 2015-10-02

**Authors:** Sabrina B. L. Araujo, Mariana Pires Braga, Daniel R. Brooks, Salvatore J. Agosta, Eric P. Hoberg, Francisco W. von Hartenthal, Walter A. Boeger

**Affiliations:** 1 Laboratório de Ecologia Molecular e Parasitologia Evolutiva, Universidade Federal do Paraná, Caixa Postal 19073, Curitiba, PR 81531–980, Brazil; 2 Departamento de Física, Universidade Federal do Paraná, Caixa Postal 19044, Curitiba, PR 81531–980, Brazil; 3 Center for Environmental Studies and Department of Biology, Virginia Commonwealth University, Richmond, VA, United States of America; 4 US National Parasite Collection, US Department of Agriculture, Agricultural Research Service, BARC East No. 1180, Beltsville, MD, United States of America; 5 Pós-Graduação em Ecologia e Conservação, Setor de Ciências Biológicas, Caixa Postal 19031, Curitiba, PR, 81531–990, Brazil; University of Minnesota, UNITED STATES

## Abstract

Despite the fact that parasites are highly specialized with respect to their hosts, empirical evidence demonstrates that host switching rather than co-speciation is the dominant factor influencing the diversification of host-parasite associations. Ecological fitting in sloppy fitness space has been proposed as a mechanism allowing ecological specialists to host-switch readily. That proposal is tested herein using an individual-based model of host switching. The model considers a parasite species exposed to multiple host resources. Through time host range expansion can occur readily without the prior evolution of novel genetic capacities. It also produces non-linear variation in the size of the fitness space. The capacity for host colonization is strongly influenced by propagule pressure early in the process and by the size of the fitness space later. The simulations suggest that co-adaptation may be initiated by the temporary loss of less fit phenotypes. Further, parasites can persist for extended periods in sub-optimal hosts, and thus may colonize distantly related hosts by a "stepping-stone" process.

## Introduction

Co-speciation has been considered the most important process governing the distribution of symbionts, especially host-parasite systems. Hence, the study of the evolution of host-parasite associations has been driven by coevolutionary frameworks in which host switching should be rare (e.g., [1]; see [2,3] for a more extensive discussion). Parasites, however, are Darwinian systems having their own evolutionary capabilities and are not simply passive followers of their host evolutionary history [2,4,5]. As a consequence, although parasites are ecological specialists with respect to some aspect of host biology, host switching has been far more common in shaping symbiotic associations than considered in the past [2,6]. Published empirical examples showing high levels of host switching include symbiotic interactions ranging from host-parasite [6–8] and plant-insect [9] systems, to microbial pathogens [10] and even brood parasitism [11,12].

The process of host switching incorporates several stages thought to be necessary for a new association to become established. These (modified from [13,14]) comprise: (i) Opportunity: The parasite must have opportunity to host switch. The potential associates must co-exist and enter in contact, temporally and spatially. Opportunity may be increased, for instance, by increasing the number of times parasites are exposed to a single new host (e.g., propagule pressure); (ii) Compatibility: To establish long-term associations, parasites and hosts must be minimally compatible. The parasite must be able to overcome barriers to establishment imposed by the host to initiate colonization of the new host resource. The barrier can be anything from specific or generalized immune responses, to physical barriers (e.g. epidermis, exoskeleton, ingestion, insertion by a vector) that must be surmounted for the parasite to reach the region of the host adequate for parasite survival. Further, a compatible host is any organism that provides an adequate resource for the parasite species both as a substrate and food-source. This includes the capacity of spreading between host individuals (i.e. parasite transmission dynamics compatible with the host's biology). Finally, mortality in the host population, as a consequence of the new association, should not compromise survival of the species involved; and (iii) Conflict resolution: The associates should resolve subsequent conflicts emerging from the basic dynamics of “living-together”. This is called *co-accommodation* [15] or *co-adaptation* (for a summary see [5]). This phase comprises evolutionary processes resulting in unilateral or reciprocal adaptations associated with the coexistence of host and parasites.

It seems un-problematical to assume that the fitness of a parasite in a newly established host association is initially sub-optimal relative to the original (source) ancestral host association, with which the parasite shares some evolutionary history. It is thus also reasonable to expect that adding a new host to the parasite repertoire would require special circumstances. Those special circumstances are conventionally assumed to be the evolution of novel genetic information allowing survival in the new hosts [10,16–18]. Yet, host shifts and host range expansions can happen more rapidly than one might expect if specific novel genetic information must arise randomly within the parasite population with respect to the potential new host [19,20].

The scenario above defines the *parasite paradox–*co-adaptive specialization should mitigate host switching and yet host switching occurs often and rapidly in real time and in evolutionary time much coevolutionary diversification has involved host switching. Agosta et al. [6] proposed a mechanistic framework for resolving the parasite paradox, beginning with *ecological fitting* [21] as the main mechanism initiating host-switching. According to Agosta and Klemens [22], parasites can incorporate new hosts by (a) colonizing a new host species that represent a very similar resource as the ancestral host, i.e. ecological fitting via resource tracking, or (b) colonizing hosts that represent new resources. i.e. ecological fitting via "sloppy fitness space". Agosta and Klemens defined "sloppy fitness space" as the region of the "fitness space" (FS) of a given species that is outside the range of conditions in which the species evolved, and yet has positive fitness. Phenotypic plasticity, correlated trait evolution, and phylogenetic conservatism all contribute to potential host switching abilities of parasite species, no matter how specialized [6,22].

Ecological fitting and sloppy fitness space are elements of the Stockholm Paradigm [23], a proposed framework for understanding the evolution of interspecific ecological associations. The Stockholm Paradigm incorporates the following concepts and processes: *Ecological Fitting* [21], the Oscillation Hypothesis [24], the *Geographic Mosaic Theory of Coevolution* [19], and the *Taxon Pulse* [25].

We postulate that the extent of the sloppy fitness space of a certain population or species varies through time. While some ecological processes may be involved in its reduction (e.g., a processes analogous to genetic bottleneck) others would be associated with its expansion (e.g., accumulation of variability through anagenesis). The resulting pattern would show cyclic changes of reduction and expansion/recuperation of the extent of the FS. The expected cyclic evolutionary pattern of the FS, we predict, should directly influence the ability of a lineage of parasitic organisms to host switch or expand its host range. If this is true, the high prevalence of host switching in evolutionary time, and of host switches among distinct host species in ecological time, become baseline expectations.

To assess these predictions, we developed an individual-based model in which the phenotypes of parasite individuals are explicitly modeled and determine fitness in their hosts. The FS is modeled as the phenotypic amplitude of a parasite lineage in a certain resource/host. Through time parasites can reproduce, die and disperse. Reproduction incorporates mutation and recombination, allowing the growth of population FS. Mortality incorporates the selection pressure imposed by the hosts and limits the growth of FS. Random hosts are offered at each parasite generation, allowing parasites to attempt dispersal to and colonization of the new host (host-switch). Our model assumes the parasite species has a single opportunity to colonize each host offered, to avoid the influence of multiple/continuous dispersal (this factor is being explored in a new model, under development). Thus, the parasite is not previously exposed to selection pressure represented by the new host resource, as at each parasite generation, new random hosts are present. As a result, our model explores the potential of host switches for a parasite species with variable sizes of its FS, presented to new and arbitrary host resources. While the stimulus for this model was host-parasite associations, our model is also adequate to test hypotheses and discover patterns of host-switching for most symbiotic associations. We assume that hosts and parasites, hosts and pathogens, hosts and brood parasites, and host plants and phytophagous insects are evolutionarily analogous symbiotic associations [26] (see [5] for a review).

Our simulations tested the above hypotheses and predictions, but also revealed other components of host switching that are compatible with published empirical studies. These include: 1. the role of propagule pressure and FS in host switching success; 2. a non-linear relationship between host switching and size of the FS; 3. survival of parasite populations at sub-optimal adaptive regions (in relation to the host); 4. a mechanism that allows host switching to occur among hosts that represent highly divergent resources.

## Materials and Methods

The model was initially based on the biology and life cycle of the Gyrodactylidae (Platyhelminthes, Monogenoidea). Gyrodactylids are mainly ectoparasitic on fishes, have a monoxenic/direct life cycle, and are capable of colonizing new hosts as adults, contrary to most parasitic platyheminthes [7]. Accordingly, the modeled parasite is assumed to have a direct life cycle, i.e. with no intervention of intermediary host or vectors and a single individual is capable of leaving offspring even in the absence of a partner. However, as we mentioned previously, we believe that the model is applicable to understanding similar processes in most symbioses, including heteroxenic associations (including more than one species of host in the life cycle) [27–29].

**Fig 1:**
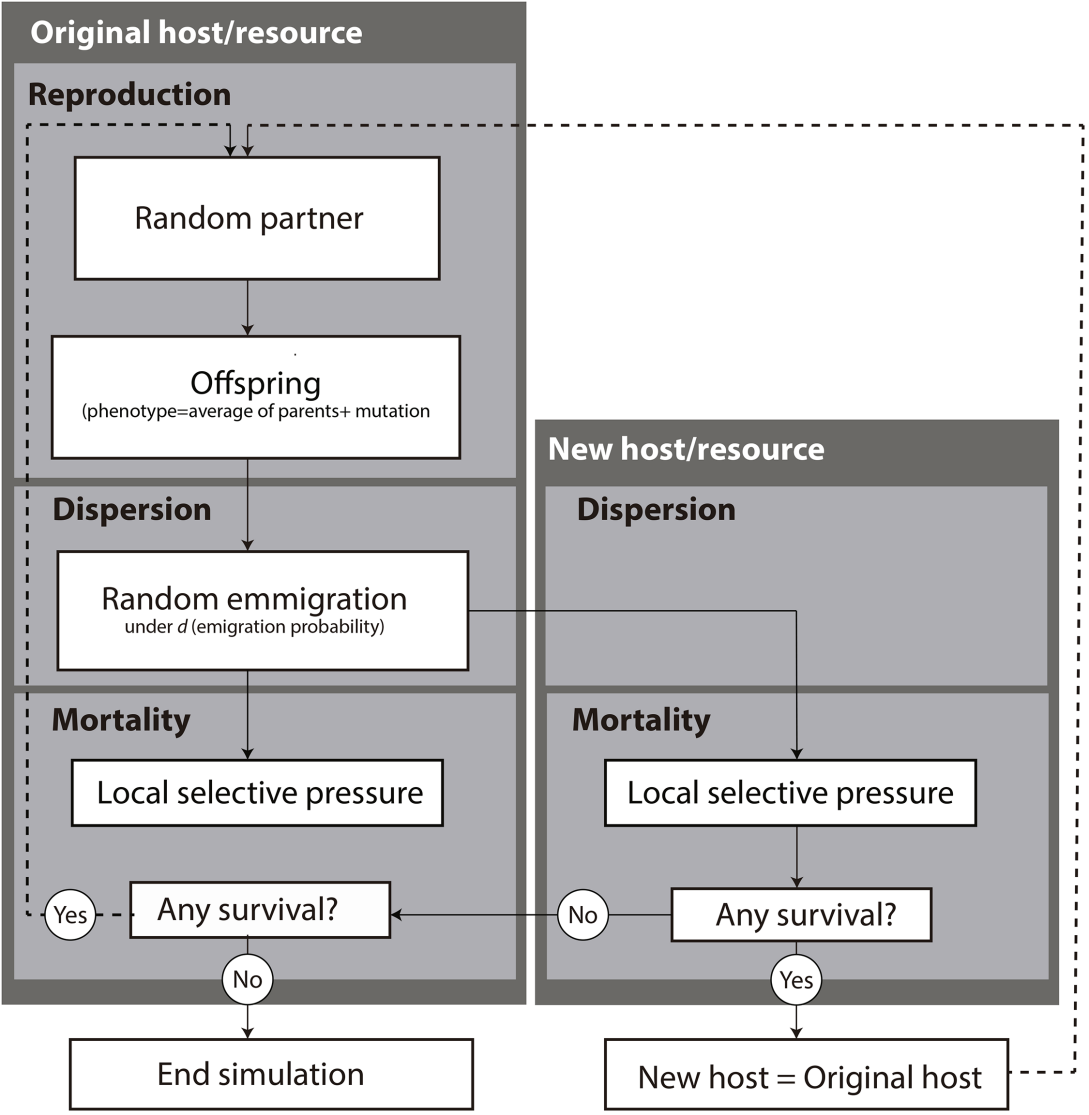
Flowchart of the model. In each generation, every parasite has a chance to reproduce, disperse to a new host, and die due to the selection pressure imposed by its respective host. For simplicity, after each case of successful host-switch, the parasite population on the ancestral host is no longer modeled and the “new host” becomes the “original host”. The simulation stops when all individuals die.

We evaluate the extent of host switching of a parasite lineage under variable Fitness Space using an Individual Based Model (parasite individuals are explicitly modeled). Each parasite expresses a phenotype that is defined by a value of relative fitness to a host resource. The model dynamics emerge by offering different hosts to the parasites and imposing reproduction, dispersion and mortality events (Fig 1). A program was designed to run the simulations (versions executable in Windows, Linux, and MacIntosh are available at http://fisica.ufpr.br/araujosbl).

For simplicity, we assume that the extent of FS is associated solely with accumulated phenotypic variation emerging from mutation and recombination during reproduction; we do not explore the fraction of the FS resulting from phenotypic plasticity, phylogenetic conservatism, or exaptation (as suggested by Agosta and Klemens [22]). Hence, we opted to use the term Information Space (IS) instead of FS. The IS represents solely a small portion of the inheritable properties of the parasitic organism being modeled, only those that may influence the fit of the organism to the host resource being considered. Parenthetically, if plasticity, phylogenetic conservatism, and exaptations were included as additional variables in our model, phenotypic variability could be greatly increased, but never decreased.

Thus, the IS of a population/species is the amplitude of the population phenotypic space. The phenotype of each parasite (*i*) is explicitly modeled and represented by a real number, *p*_*i*_ (its relative fitness to a host species). Individual fitness is a function of the proximity between the phenotype of the parasite individual and the optimum phenotype for a given resource (detailed below).

**Fig 2:**
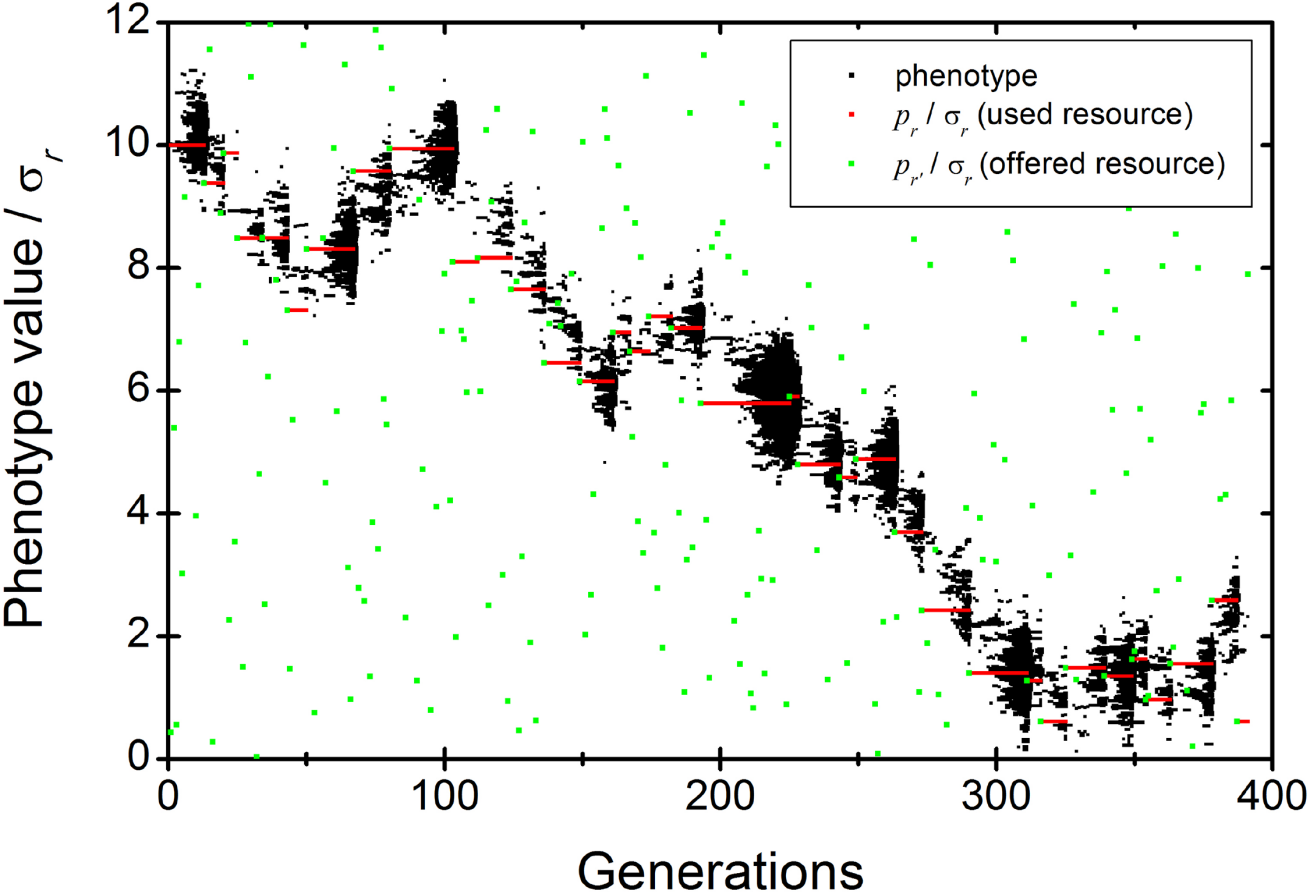
Temporal evolution of the information space of the parasite population. The black dots represent all different population phenotypes. The red line is the optimum phenotype value favored by the colonized resource (*r*). The green points represent the optimum phenotype favored by a new resource (*r*') offered at each new generation. When a new resource is successfully colonized, the source population isn’t plotted any more (the end of a red line means an extinction only for the last generation). Parameters used in this simulation are listed in Table 1.

All simulations start with an initial parasite population composed of a single individual (*N*_*0*_ = *1*) using a resource with an arbitrary value of phenotype optimum (p_*r*_ = *5*). To avoid extinction in the first simulation steps, we impose the best-adapted initial population, p_*i*_ *=* p_*r*_ = *5*. Each simulation runs until population extinction, or for 1000 generations (Fig 2). At each parasite generation, a new resource (*r*’), for which the phenotype optimum value p_*r’*_ is randomly defined and made available to the parasite population.

In each generation, all individuals of the parasite population have a chance to reproduce, disperse to *r*’, and die. Surviving individuals and their offspring generated at time *n* form the population at time *n*+1, which goes through all events again, composing the population at time *n*+2 and so on, until a successful host-switch occurs. Again for simplicity, after each case of successful host-switching, the parasite population on host *r* (the ancestral host) is ignored by the model (i.e. no data on the original parasite population on that host is further gathered) and the simulation follows the new founding population on *r*’ (Fig 1).

### The host/resource

Individual fitness is modeled by a survival probability in the resource, which follows a normal distribution centralized in |*p*_*i*_ *—p*_r_|, i.e. the phenotypic match of the parasite to the given resource:

$$P\left(p_{i},p_{r}\right)=\text{exp}\left[\frac{{\left(p_{i}-p_{r}\right)}^{\text{2}}}{{\text{2σ}}_{r}^{\text{2}}}\right]$$ (1)

where σ_r_ is the standard deviation. Thus, parasites whose phenotype matches the resource optimum perfectly have maximum survival, and survival decreases with increasing distance from the optimum phenotype. This is the essential ingredient that allows individuals with less-than-perfect fit to survive; a fundamental element of Darwin’s theory of natural selection [30]. In this model, we assumed that parasite-induced host mortality does not influence population growth and phenotypic diversification.

### Reproduction

At each time step of the cycle, the population can reproduce with a birth rate of *b* offspring per individual. However, we assume that the total population cannot exceed the carrying capacity *K*. Parents are chosen at random, with repositioning of the first chosen parent into the second draw, allowing self-fertilization. Offspring phenotypes are equal to the arithmetic average of their parents phenotypes, plus a variation δ, which value is a random number that follows a normal probability function:

$$P\left(\text{δ}\right)=\text{exp}\left[\frac{{\text{δ}}^{\text{2}}}{{\text{2σ}}_{v}^{\text{2}}}\right]$$ (2)

where σ_*v*_ is the standard deviation. The arithmetic average and the random variation represent evolutionary novelties that originate during reproduction by sexual recombination and spontaneous mutations, respectively.

The "daughter" phenotype may be more or less fit than the "parental" phenotypes in relation to a specific resource, but can persist so long as fitness is greater than zero. A single unchanged selective pressure (single, invariable host species) is modeled and represents the synthetic fitness to a specific host species.

### Dispersion and Mortality

As mentioned, in each generation, a new resource *r*’ is made available. The optimum phenotype favored by this resource, *p*_*r'*_, is a uniform random real number within the range *p*_*r*_ *- δ*_*r*_ *≤ p*_*r'*_ *≤ p*_*r*_ *+ δ*_*r*_, where *δ*_*r*_ is a parameter that limits the maximum amplitude of the new resource. Each individual has probability *d* of dispersing from the resource *r*, thus, the amount of opportunities to host switch is proportional to the population size. Those individuals that attempt dispersal can colonize the new resource following the survival probability in the new resource *r*', *S(p*_*i*_,*p*_*r'*_ *)* Eq (1). In each generation, mortality can occur even if a dispersal attempt fails, but here the survival probability is calculated over *r* instead of *r*', *S(p*_*i*_,*p*_*r*_ *)*, Eq (1).

### Success in host-switching

A successful host-switch is a colonization event that does not lead to population extinction; conversely, if all the parasites in the new resource die (regardless of the generation in which it occurs), it is a failed host-switch attempt. Fig 2 shows a sequence of successful host-switches, except the last one, which leads to population extinction.

### Data analysis

For a given set of parameters (see Table 1), we investigated the probability of successful colonization by host switching as a function of: (i) size of the population; (ii) time (in terms of number of generations) between two consecutive host switches; (iii) size of the information space, IS = *(p*_*max*_ *- p*_*min*_ *) / σ*_*r*_; (iv) absolute phenotypic distance between the resource being used and the new one, *|p*_*r*_ *- p*_*r'*_ *|/ σ*_*r*_; (v) absolute distance between the resource in use and the midpoint of population phenotypic distribution, *|p*_*r*_ *−IS*_*p*_ *|/ σ*_*r*_, where *IS*_*p*_ = *p*_*min*_ *+IS/2*; (vi) maximum distance between parasite phenotype and the resource *|p*_*r*_ *- p*_*max*_ *|/ σ*_*r*_. We also estimated the Spearman’s partial correlation between population size and IS with colonization time as the controlling variable.

**Table 1:** Short description of model parameters (with the values imposed in all figures) and variables.

Parameter	Short meaning
*N*_*0*_ = *1*	Population size when the simulation starts
*b = 0*.*5*	Birth rate
*K = 500*	Carrying capacity
*d = 0*.*05*	Emigration rate
*σ*_*r*_ = *0*.*5*	Standard deviation of the survival probability function (Eq 1)
*σ*_*v*_ = *0*.*2*	Standard deviation that defines phenotypic variation (Eq 2)
δ_*r*_ = *5*	Maximum distance between two consecutive resources
Variables	
*p*_*r*_	Population optimum phenotype favored by the resource *r*
*p*_*r'*_	Population optimum phenotype favored by a new resource *r*’
*p*_*i*_	Phenotype of an individual *i*
IS	Information space at a given generation

For these calculations we iterated the system during 1000 generations or until an extinction event occurred. In order to avoid a transient effect, the dynamics from the 1st to the 50th generation were excluded from the analysis. As the migration amplitude is directly affected by standard deviation of the survival probability function, all terms that involve calculation of distance (iii–vi) were standardized by this value.

## Results

We investigated the robustness of our results considering different birth rates (b = {0.5, 2}), carrying capacity (K = {500, 1000, 5000}), emigration rate (d = {0.05, 0.1, 0.2}), and standard deviation that defines phenotypic variation (*σv* = {0.1, 0.2}). The qualitative results agree among all tested combinations of parameters. Although the choice of the parameters was made arbitrarily (we did not use any real data to fit them) all parameter combinations have the same qualitative results. Herein we present the outcomes for a representative set of parameters (present in Table 1).

**Fig 3:**
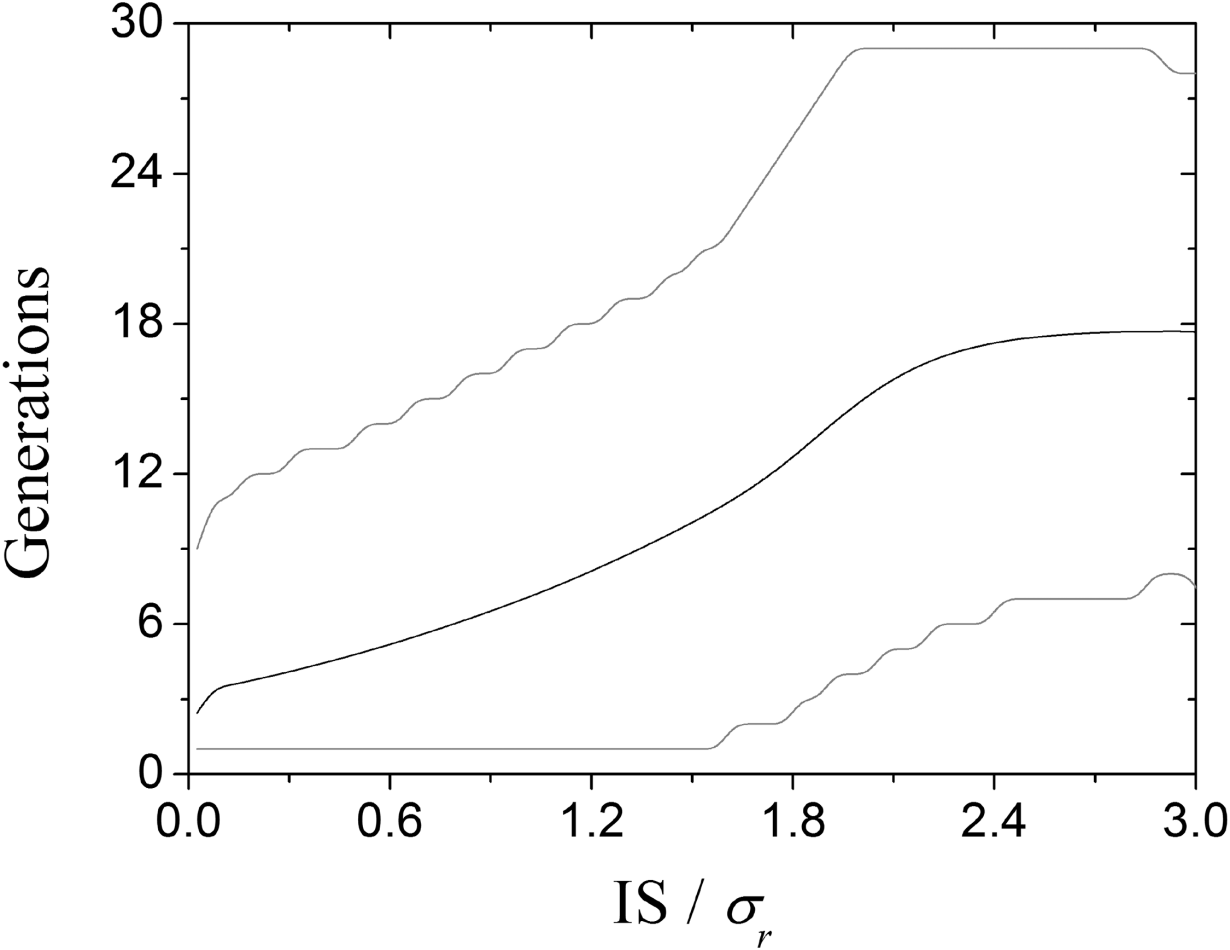
Relation between the size of IS and the number of generations in a specific host just before a successful colonization. Black and gray lines show the mean and the 95% confidence interval, respectively. This graph considers 10^9^ repetitions of the parameter values listed in Table 1.

**Fig 4:**
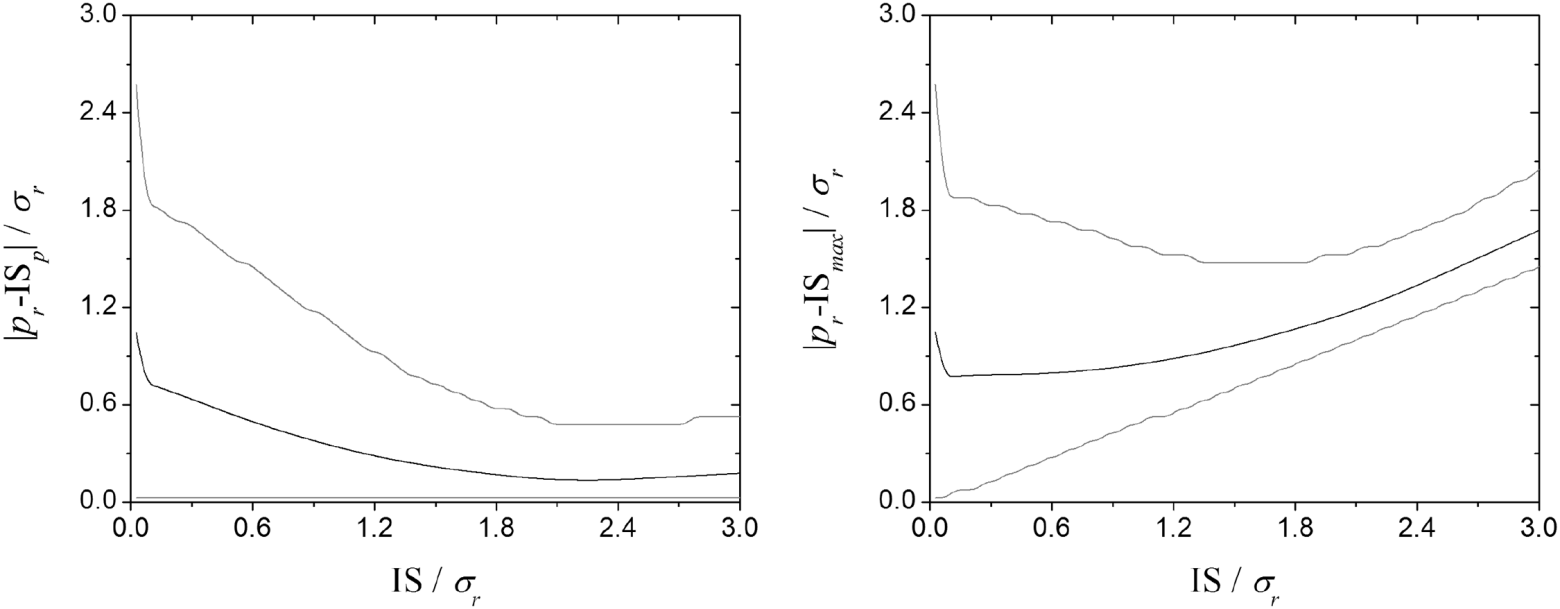
Midpoint of IS (a) and its maximum amplitude (b) in relation to the utilized resource as function of IS. Black and gray lines show the mean and the confidence interval (of 95%), respectively. When the population is distant from the optimum (the superior confidence curve in both graphs), as the IS increases, the population evolves towards the optimum phenotype imposed by the host (a) and also loses its maximum amplitude (b) suggesting that the increasing of variation does not compensate co-adaptation (for *IS/ σ*_*r*_ *<1*.*5*). However, as population becomes more co-adapted, the maximum amplitude of IS recuperates. These graphs consider 10^9^ repetitions of the parameters values listed in Table 1.

**Fig 5:**
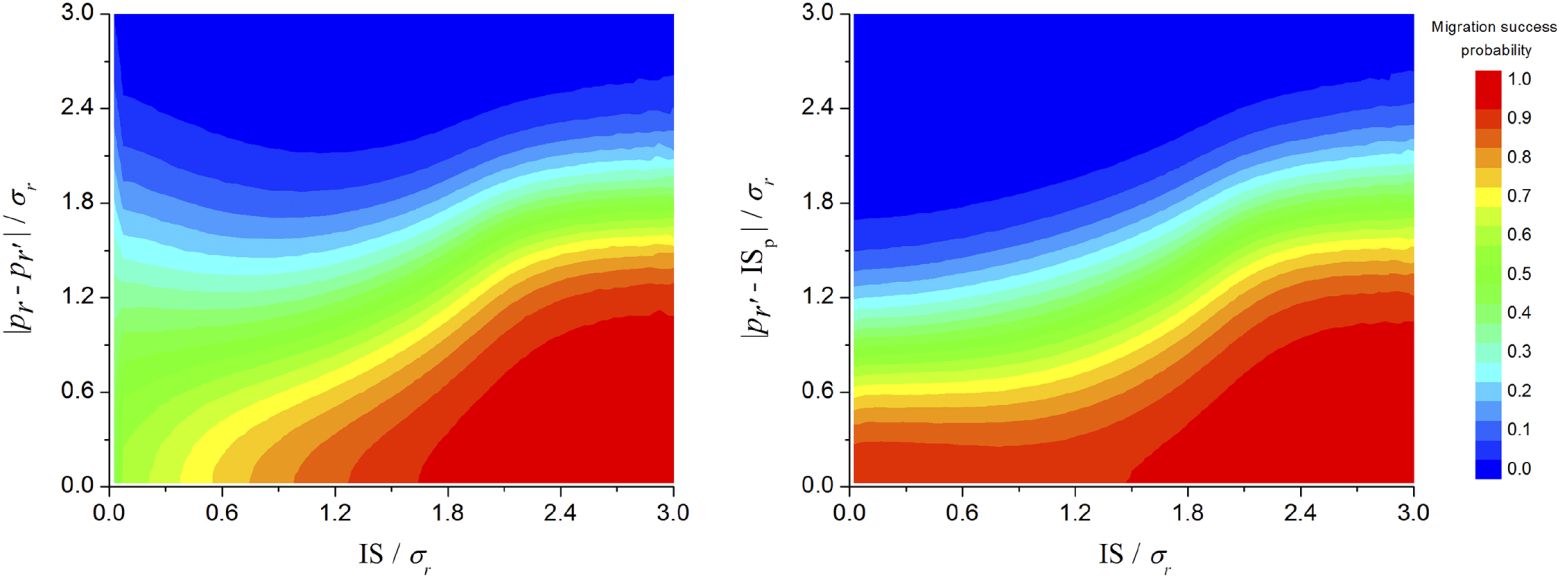
Phase diagrams. Each diagram shows the probability of successful colonization (color legend at right) as function of information space (IS) and (a) absolute distance between colonized resource and the new available one; (b) absolute distance between the new resource available and the midpoint of population phenotype distribution. This graph considers 10^9^ repetitions of the parameters values listed in Table 1.

Despite the small initial size of the IS in each independent simulation (*N*_*0*_ = *1*, IS = *0*) and the selective pressure imposed by the host resource, reproduction incorporating "mutation" and recombination produces rapid growth in a population’s IS along with increase in population size (Fig 2). The overall dynamics are as follows: a limited number of individuals (dispersers) colonize the new resource, so the IS is initially small; then reproduction of sequential generations increases the IS (Fig 3) while differential mortality of composite phenotypes directs the IS towards the selective optimum of the host resource. In some cases, a parasite population persisted for more than 10 generations with small IS (Figs 2 and 3) located some distance from the selective optimum (Fig 4). Considering the space of available resources simulated, host switches occurred between hosts within a distance not greater than 3σ_r_ (Fig 5a). In fact, from Eq (1), the probability that an individual survives at distance 3σ_r_ is about 1%. More distant resources were reached with sequential host switching; observe in Fig 2 that the sequential host switching changed the used resource from *p*_*r*_ */σ*_*r*_ = *10* to *p*_*r*_ */σ*_*r*_ *E1*, that is about *9σ*_r_.

**Fig 6:**
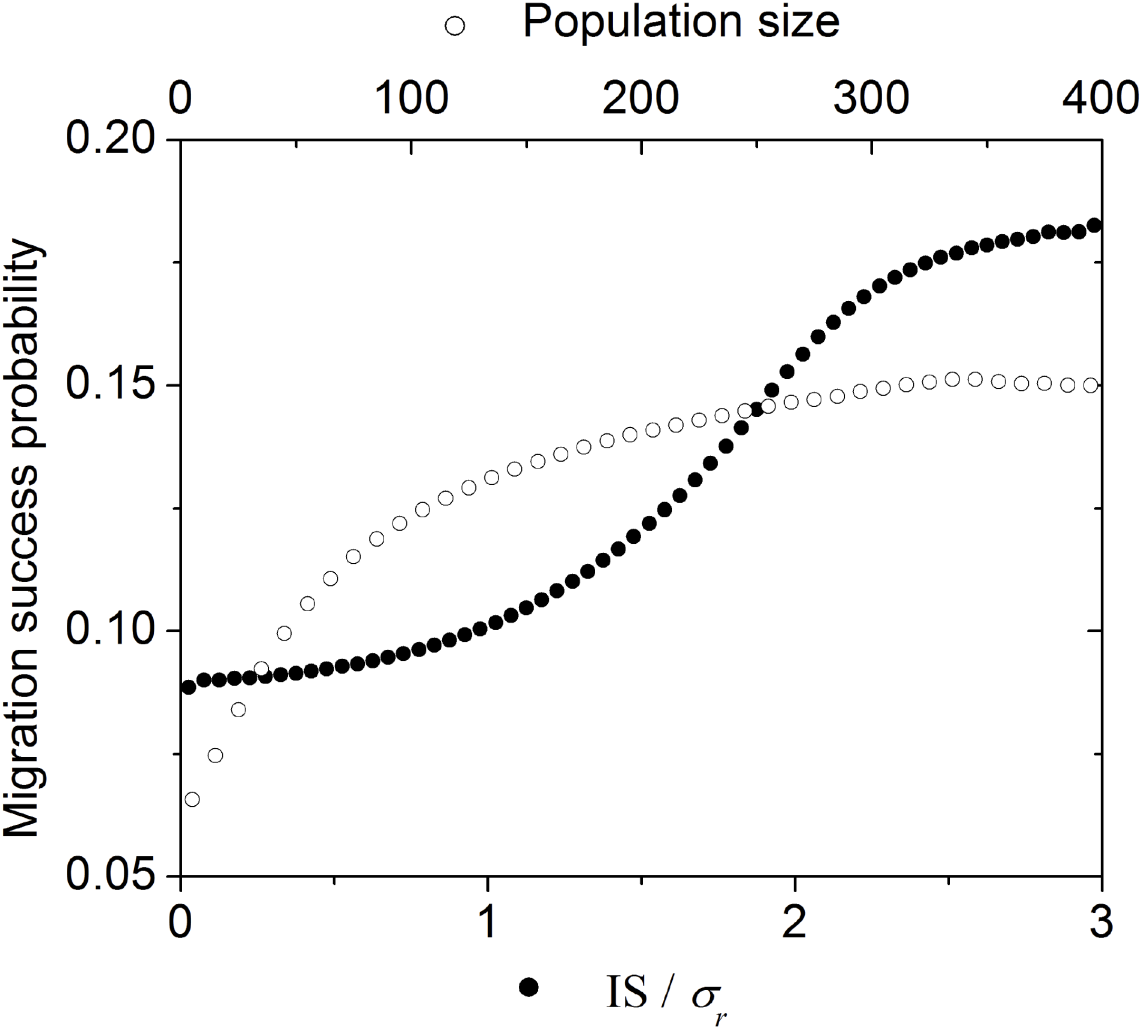
Effect of information space amplitude and population size on migration success probability. This graph considers 10^9^ repetitions of the parameters values listed in Table 1.

Population size and IS are correlated independently of the colonization time (Spearman’s correlation coefficient: 0.89, p<0.01). Host-switching success is associated both with the IS size and the population size (Fig 6), but not in the same circumstances. While IS expansion increases the range of possible new resources, the increase in population size increases the probability of dispersal events, as each individual has a probability *d* of dispersing. However, when a population exceeds a certain size (about 200 individuals in our model space) there is a limited and almost negligible increase in the probability of host-switching success with increasing population size (Fig 6). Allowing the phenotypic variation of the increasing population to escalate results in greater success of host switching, supporting our initial decision not to include phenotypic plasticity as an explicit variable.

The probability of host-switching and the size of the IS are not related linearly (Figs 3 and 4). Considering the phenotypic distance between the old and the new resource (*|p*_*r*_ *- p*_*r'*_ *|/ σ*_*r*_) (Fig 5a), even with very low values of IS (IS<0.4*σ*_*r*_), parasite populations could reach fairly distant new host resources (e.g. 0.3−0.4 migration success probability at distances up to 1.5*σ*_*r*_). When IS is small (IS<1.0*σ*_*r*_), the relationship between IS and success varies with the distance between resources: success increases with IS for shorter jumps, and decreases with IS for longer jumps (Fig 5a). Indeed, host-switching success is not directly dependent on the distance between resources, but rather on how much of the new host resource was encompassed by the parasite population just before the switch. Even populations of parasites with extremely small IS on the original resource are 100% successful in colonizing close resources (i.e. at distances of up to 0.3*σ*_*r*_) (Fig 5b).

In populations with small IS, the midpoint of the IS generally does not coincide with the resource optimum (Fig 4a) because these populations originate from recent host-switching events (Fig 3). Following a colonization event, the IS of the population in the new host increases and evolves towards *r* (Fig 4a). Initially the evolution towards *r* is faster than the increase of the IS and *p*_*max*_ decreases, subsequently the evolution towards *r* decelerates and *p*_*max*_ increases (Fig 4b).

## Discussion

Our model is based on two properties critical to the evolutionary process: acquisition of evolutionary novelties (e.g. by mutation and recombination) and relative fitness (survival probability on a host, Eq 1). This represents a minimalistic-approach model; thus, the results presented herein show that host switching can occur readily in lineages/populations with variable phenotypic amplitude, even if they are highly specialized ecologically. Our results support six main conclusions described below.

First, as predicted by ecological fitting [6,20,21], host colonization (increasing host range) does not require prior evolution of novel genetic information associated with the fitness of the parasite population in the new host species. The major factors influencing the success of host switching are compatibility and opportunity. Compatibility is proportional to the relative fit of the species involved, represented in our model by a host whose selective pressure is not strong enough to eliminate the colonizing parasite population. Opportunity—the possibility of physical contact in time and space of potential associates—emerges from geographic distributions and trophic structure [2,31].

Second, as anticipated on theoretical grounds, our simulations produced cyclic changes in the size of the IS. These are associated directly with the change in the potential to host switch to distinct host resources (Figs 1 and 5). Following a host-switch, only a portion of the original parasite population is retained in the new host resource, resulting in a reduction in the size of the new population IS, similar to a genetic 'bottleneck' effect [32]. Recombination and the emergence of evolutionary novelties ('mutations' in the model) accumulate composite phenotypes with corresponding relative fitness to the host species, increasing the IS of the parasite population in the new host. Growth of the IS thus is limited by the host selective pressure. The *Oscillation Hypothesis* [24]–a critical element of the Stockholm Paradigm—predicts that colonizing specialists will become generalists, and those generalists will then produce new isolated specialists. We are currently investigating the possibility that the oscillating behavior in IS uncovered in this initial model can be linked directly to the Oscillation Hypothesis.

Third, successful colonization of new hosts is not simply a matter of propagule pressure (i.e., the amount of opportunities to host switch); the dynamics of host switching success involve both parasite population size and IS (Fig 6). Every organism has equal probability of attempting host switching, so propagule pressure is directly proportional to the size of the original population. Immediately following a colonization event (when the population is in a sub-optimal condition), population density and IS decrease. At this point, increasing population size has a strong influence in colonization success (see the curve slope in Fig 6). As the population increases, the gain in colonization success becomes less and less dependent on population size but still dependent on IS. Thus, success in host switching is not driven solely by propagule pressure (i.e., quantity), but also by the amount of collective information (within the original IS) these propagules represent. This makes intuitive sense since, as discussed above, successful responses to ecological novelties require both opportunity and compatibility. Ecological fitting via resource tracking can be accomplished by any given parasite population, whenever the new and the ancestral hosts represent similar resources and selective pressures (ecophysiologically equivalent hosts, see [27–29]). On the other hand, ecological fitting via sloppy fitness space is directly related to the IS of the parasite population, so that host-switching success increases with increasing IS. Ahlroth et al. [33] (also Lockwood et al. [34]) documented a similar pattern for invasive species. They suggested that propagule pressure is associated with the amount of genetic variation in the introduced population, improving the chances that the invasive population will be able to adapt successfully to novel selection pressures in the recipient location. Large-scale or long-term propagule pressure influence the success of invading new and habitats (similar to host switching) because it is directly coupled with the extent of the original IS/FS of the colonizing population.

Fourth, the relationship between host-switching success and amplitude of IS varies with the functional (i.e., evolved) distance between resources. Although host-switching success generally increases with increasing IS, when IS is small host-switching success between distant resources decreases with IS (Fig 5a) due to the interaction between increasing variability and selection. Our model recognizes that the colonizing founder population associated with a new host not only may originate, but may also persist far from the resource optimum. The farther from the resource optimum, the stronger the selective pressure for the colonizing population to evolve toward the optimum point. The changes in population phenotypic distribution occurring during this phase refer to co-accommodation [15], which includes possible co-adaptive responses. Our model indicates that co-accommodation may occur more rapidly than the increase in variation if the parasite population is far from the fitness optimum of the new host (Fig 4). This means that immediate subsequent generations of the colonizing population might have reduced ability to switch to distant resources (Figs 4b and 5a). Such a loss might be temporary; as the colonizing population evolves towards the optimum, the rate of co-accommodation should slow while variation continues to increase, as non-optimal phenotypes accumulate. This implies that opportunity and compatibility are more important than conflict resolution in establishing new host-parasite associations.

Fifth, the model shows how populations can explore the host fitness space, increasing the ability to switch to hosts that are not similar to the original host. This can be accomplished by small populations with sub-optimal fit and by large populations with large IS. Entire parasite populations with sub-optimal fit to the host were able to persist for a large number of generations (Fig 2). While the survival of individual parasites with sub-optimal fit is assumed in the model, the survival of an entire population comprising such individuals for several generations is an outcome of the dynamics, previously predicted by Agosta and Klemens [22] and Agosta et al. [6]. Small populations with low fitness may be able to switch between distant resources precisely because they are not optimally adapted to the ancestral host. Survival of a population at a sub-optimal fit in relation to the available resource has been empirically demonstrated recently. Li et al. [35] and Xue et al. [36] indicate that the giant panda, *Ailuropoda melanoleuca*, though subjected to selective pressure associated with its main resource, bamboo, for the last 7 millions years, retains an ancestral, fundamentally carnivore, digestive system. The giant panda "did not evolve any enzymes for bamboo digestion, and it still retains all necessary enzyme homologs for a carnivorous digestive system" [35]. Furthermore, Xue et al. [36] indicated "the giant panda appears not to have evolved a gut microbiota compatible with its newly adopted diet, which may adversely influence the coevolutionary fitness of this herbivore".

Sixth, our model shows that parasites may colonize hosts by a "stepping-stone" process [37], accessing hosts that are quite divergent from each other in fitness space by host switching events involving hosts that are in relative proximity (see Fig 2). Such processes could result from host switches among sympatric species, sequential geographic overlap among hosts colonized during the biotic expansion phase of a taxon pulse, or from changes in trophic structure in conjunction with, or independent of, geographic expansion. Stepping-stone dynamics have been implicated for emerging disease dynamics, on historical and contemporaneous time scales. For example, Gorman et al. [38,39] (see also [40]) provided evidence supporting the hypothesis that the Influenza A virus originated in birds. Subsequently, swine acquired the virus, which differentiated into a swine form. Human Influenza A is apparently a derivative of the swine form. Swine remain suitable hosts for all three forms of the virus. Swine infected with avian and human, or swine and human Influenza A provide the possibility of genetic exchange producing strains highly pathogenic to humans. Braga et al. [37] recently showed that this stepping-stone process has strongly influenced the composition and sharing of genera of Monogenoidea (Platyhelminthes) among lineages of freshwater fishes in the Neotropics.

Our model attempts to understand host switching at the lowest level of complexity possible. Host colonization that does not require correlated genetic change provides formal recognition of ecological fitting and its significance relative to issues of host range dynamics and host switching by ecological specialists [5,6,20]. Such a minimalist model, however, does not consider complications such as implicit host variability, other components of FS (as proposed by Agosta and Klemens [22]), continuous dispersal and genetic exchange of parasite populations on distinct host species and host mortality, among others. Other components of the Stockholm Paradigm (the Oscillation Hypothesis, the Geographic Mosaic Theory of Coevolution and the Taxon Pulse hypothesis) remain to be evaluated by future studies. In particular, we expect that the cyclic changes in IS/FS, revealed in the present simulations, could provide direct evidence for the host-range dynamics proposed by the Oscillation Hypothesis.

According to the model, large populations can explore host fitness space extensively by accumulating non-optimally adapted phenotypes, which increase the capability of the population to use a larger variety of hosts (Fig 5). A fundamental Darwinian view is that variants well-suited to existing local conditions dominate numerically, but variants with low (non-zero) fitness are more likely to survive changes in those conditions [5,41]. Extinction of an optimal host thus will not necessarily result in extinction of a parasite lineage, even if the parasite is highly specialized on that host (*true specialist* of Brooks and McLennan [5]) or has had no opportunity to interact with other hosts prior to the episode of environmental perturbation (*faux specialist* of Brooks and McLennan [5]). The discovery of parasite lineages that are much older than current host lineages and in which persistence and diversification have resulted from episodes of host colonization indicates this is a non-trivial aspect of parasite evolution [2]. Documented cases of helminths inhabiting vertebrates in terrestrial and marine systems comprise episodes of retro-colonization, lineage persistence, and novel diversification initiated by host switching across ecologically equivalent groups, before or during episodes of global-scale extinction and ecological perturbation [42–47]. These episodes of host colonization have been the drivers for persistence of major parasite lineages on evolutionary and ecological time scales [48–50].

This wealth of empirical findings, in conjunction with the simulation results presented herein, suggest novel insights into the complex phenomena associated with emerging infectious diseases. For example, we must be careful about assuming that newly-documented strains of emerging pathogens are the result of new mutations associated with, or caused by, host switches. Our model suggests that such changes are unnecessary for host switches to occur, and that host switches into sub-optimal hosts may involve rare but pre-existing phenotypes, difficult to sample in native populations of the parasite. The working assumption that any parasite, even a virus, is genetically monomorphic until it mutates into a form allowing it to switch to a new host may seriously under-estimate the amount of naturally occurring variation [51,52].

The Stockholm Paradigm suggests that host switching and host range expansion are more often the consequence of taking advantage of opportunities offered by a dynamic host landscape than of the evolution of novel host-use capabilities. Therefore, host switches are likely more relevant than previously considered to the evolutionary history of parasite lineages. Given opportunity, ecological fitting allows even highly specialized parasites to explore the available resources, increasing host-ranges. Phenotypic variation accumulated during periods of environmental stability enhances the ability to host-switch by ecological fitting during periods of environmental change [2]. Taken together, our simulations support empirical findings and theoretical frameworks suggesting that emerging diseases are "evolutionary accidents waiting to happen" [53] that can be anticipated, rather than rare events occurring randomly [3,23,54–56].

That a parasite population may, as suggested by our simulations, survive for many generations in sub-optimal hosts, is especially significant in planning strategies to avoid introduction of new parasitic diseases into new geographical areas. Many "reservoir" species may not be promptly recognized due to low density of the parasite population associated with its sub-optimal fit in that host. As a result, the best disease management/mitigation protocols, which may minimize exposures, would emphasize knowledge of parasite occurrence relative to hosts and geography, patterns of specialized transmission dynamics, and identification of natural or anthropocentric invasion pathways [57–60]. The expansion of human population and its correlates—agriculture and urbanization—combined with global climate change make this a daunting task [56]. Thus, for pathogens such as Ebola, one of the most critical elements is finding the natural reservoirs and assessing the range of potential non-human reservoirs (both optimal and sub-optimal host species). Any time a human infection is reported in a new geographic location (thus, evidencing opportunity), searches for infections in non-human reservoirs must occur simultaneously with public health efforts to treat the infected human host and prevent human-to-human transmission. Once established in non-human reservoirs, the pathogen may persist as a never-ending source of evolutionary experiments in host switches. And the more geographic ranges expand and trophic structures are altered–i.e. through climate change and anthropogenic activities–the more often such attempts will succeed.
